# Targeting the unfolded protein response in cancer: mechanisms, small-molecule inhibitors, and translational challenges

**DOI:** 10.3389/fimmu.2026.1878552

**Published:** 2026-07-30

**Authors:** Meizhen Lin, Zhijie Li

**Affiliations:** 1 Department of Pediatrics, Shengjing Hospital of China Medical University, Shenyang, China; 2 Liaoning Key Laboratory of Research and Application of Animal Models for Environmental and Metabolic Diseases, Medical Research Center, Shengjing Hospital of China Medical University, Shenyang, China

**Keywords:** unfolded protein response, IRE1α, PERK, ATF6, chemical chaperones, cancer therapy

## Abstract

Endoplasmic reticulum (ER) stress, triggered by the accumulation of misfolded proteins, activates the unfolded protein response (UPR) to restore protein homeostasis. Dysregulated ER stress responses have emerged as critical modulators of cancer progression and immune escape, influencing the initiation, development and maintenance of antitumor immunity. The UPR is mediated by three principal sensors—PERK, IRE1α, and ATF6—each operating at distinct regulatory levels to coordinate translational reprogramming, RNA processing, and transcriptional reprogramming. Through these mechanisms, ER stress promotes malignant progression, tumor growth, and metastasis, while excessive activation can instead trigger cell death. Given this context-dependent duality, pharmacological targeting of the UPR has emerged as a promising anticancer strategy. For instance, IRE1α inhibitors block XBP1 splicing and RIDD-mediated immune escape, PERK inhibitors and ISR modulators reverse chemoresistance, ATF6-targeted strategies modulate ATF6-dependent tumor growth and treatment responses, and chemical chaperones exhibit both cytoprotective and antitumor effects depending on tumor context. This review integrates recent mechanistic insights into UPR-driven tumor progression, including pathway crosstalk, immune regulation, and immunotherapy resistance, with advances in small-molecule inhibitors, while critically evaluating their therapeutic potential and translational challenges in cancer treatment.

## 1 Introduction

The endoplasmic reticulum (ER) is essential for protein synthesis, folding, and post-translational modification, processes that determine cellular function, fate, and survival. In various types of cancer, oncogenic, transcriptional, and metabolic abnormalities collectively create a hostile microenvironment, disrupting ER homeostasis in tumor cells (1). Various internal and external stressors, such as oxidative stress, nutrient deprivation, viral infections, and pharmacological agents, can impair ER function and trigger ER stress. This dysfunction is typically characterized by a disruption in the ER’s capacity to fold and post-translationally modify secretory and transmembrane proteins, resulting in the accumulation of unfolded proteins (2). To maintain intracellular homeostasis, ER stress activates the UPR through three transmembrane sensors: PKR-like ER kinase (PERK), inositol-requiring enzyme 1α (IRE1α), and activating transcription factor 6 (ATF6). Accumulation of misfolded proteins triggers UPR activation by displacing binding immunoglobulin protein/glucose-regulated protein 78 (BiP/GRP78) from these sensors, thereby relieving their inhibitory binding. Activated PERK phosphorylates eukaryotic initiation factor 2α (eIF2α) to transiently attenuate global protein translation while selectively promoting activating transcription factor 4 (ATF4)-mediated expression of stress-adaptive genes; however, sustained PERK signaling can induce apoptosis through C/EBP homologous protein (CHOP). Activated IRE1α undergoes oligomerization and autophosphorylation, enabling its RNase activity to mediate unconventional splicing of XBP1 mRNA to generate the transcription factor XBP1s and to initiate regulated IRE1-dependent decay (RIDD) that degrades specific RNAs to alleviate ER stress (3). Activated ATF6 translocates to the Golgi apparatus, where it is proteolytically cleaved to generate an active N-terminal fragment that enters the nucleus and activates transcription of genes encoding protein-folding chaperones and ER-associated degradation (ERAD) components (4, 5). Collectively, these pathways collaborate to bolster protein folding, clear misfolded proteins, and reduce new protein influx. However, when ER stress becomes prolonged or excessive, the UPR shifts from an adaptive program toward proapoptotic signaling. This stress-response system plays a critical role in cancer development and progression. Through downstream transcription factors including ATF4, XBP1s, and cleaved ATF6, the UPR regulates genes encoding molecular chaperones, ERAD components, and regulators of oxidative stress responses, autophagy, mitochondrial function, and cellular metabolism. The expression of these genes varies depending on tissue type and cellular context (6).

ER stress is increasingly recognized as a critical regulator of both tumorigenesis and tumor progression. ER stress in tumor cells can arise from multiple factors, including oncogenic signaling, metabolic alterations, and stress conditions imposed by the tumor microenvironment. When maintained at moderate levels, ER stress activates adaptive responses that support tumor growth, metastasis, angiogenesis, immune evasion, and resistance to therapy. However, when ER stress becomes excessive due to the persistent accumulation of misfolded proteins, it can overwhelm these protective responses and shift the UPR toward proapoptotic signaling (1, 7). Given its dual role in cancer, modulating ER stress responses has emerged as a promising therapeutic strategy. Targeting ER stress signaling not only offers opportunities to suppress tumor growth and enhance treatment efficacy but also provides a potential avenue to overcome resistance to conventional therapies. Although recent reviews have provided valuable overviews of ER stress biology in cancer and other disease contexts (8, 9), fewer studies have systematically connected UPR branch-specific pharmacology with clinical development, tumor immunity, predictive biomarkers, patient stratification, and emerging therapeutic strategies. Unlike previous broad overviews that primarily emphasize ER stress biology or general therapeutic concepts (8, 9), this review links the molecular functions of PERK, IRE1α, and ATF6 to cancer progression, pathway crosstalk, immune escape, and therapeutic resistance. We further evaluate small-molecule inhibitors targeting IRE1α, PERK, and ATF6, together with chemical chaperones, and distinguish compounds that remain preclinical from agents that have entered clinical development, such as MKC8866 and HC-5404/HC4. In addition, we discuss how UPR signaling shapes T-cell dysfunction, immune checkpoint regulation, antigen presentation, and resistance to immunotherapy. Finally, by considering predictive biomarkers, patient stratification, nanomedicine-based delivery, targeted protein degradation, and rational combination strategies, this review aims to outline practical opportunities and remaining barriers for translating UPR-targeted therapies into cancer treatment.

## 2 Unfolded protein response and tumor progression

Accumulating evidence indicates that the unfolded protein response (UPR) plays a critical role in tumor progression under persistent ER stress. Through the coordinated activities of the three ER stress sensors—PERK, IRE1α, and ATF6—UPR signaling regulates diverse processes, including tumor cell survival, metabolic adaptation, immune interactions, and therapeutic resistance. The following sections discuss the mechanistic roles of these pathways in shaping tumor development and progression.

### 2.1 PERK–eIF2α–ATF4 signaling in translational reprogramming and stress adaptation

**Figure 1:**
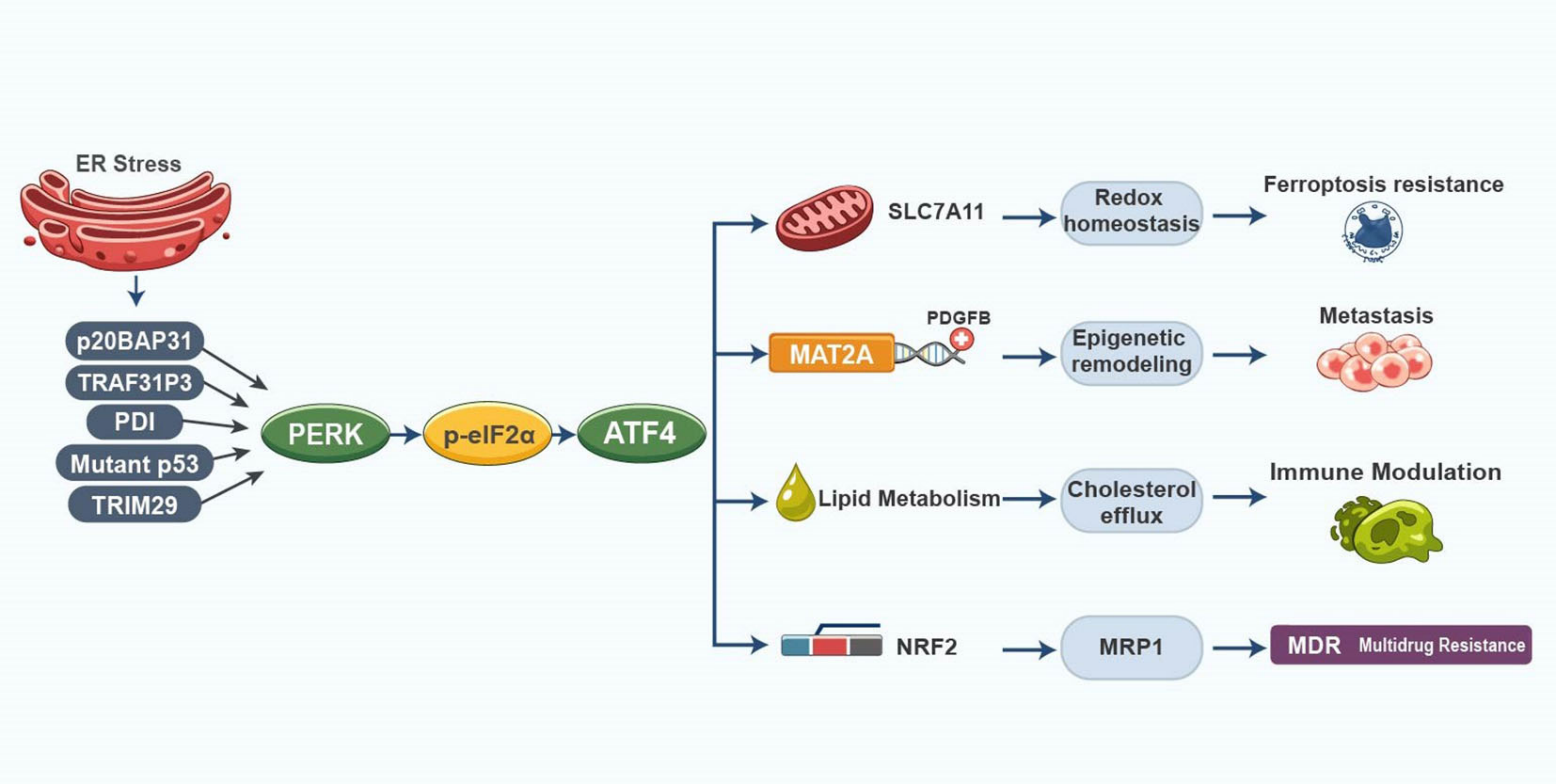
PERK–eIF2α–ATF4 signaling in translational reprogramming and stress adaptation. ER stress activates PERK, leading to phosphorylation of eIF2α and selective translation of ATF4. ATF4 regulates transcriptional programs involved in redox homeostasis, metabolic reprogramming, immune modulation, and multidrug resistance that support tumor adaptation to stress.

The PERK–eIF2α–ATF4 pathway represents a central stress-responsive module that enables tumor cells to translate ER stress signals into adaptive transcriptional and translational programs that shape tumor behavior (**Figure 1**). Upon ER stress activation, PERK phosphorylates eIF2α, leading to selective translational reprogramming and induction of ATF4-dependent transcriptional programs (10, 11).

#### 2.1.1 Molecular mechanisms underlying PERK signaling in cancer

##### 2.1.1.1 Upstream regulation of PERK activation

PERK activation can be enhanced by displacement of GRP78, as p20BAP31 competitively binds GRP78 and promotes PERK hyperactivation, thereby inducing apoptosis and significantly suppressing tumor growth through activation of the PERK pathway (12). Similarly, TRAF3IP3 promotes activation of the PERK–ATF4–CHOP cascade, leading to ER stress–associated apoptosis and inhibition of tumor cell proliferation (10). Protein disulfide isomerase (PDI) stabilizes PERK protein levels by preventing proteasomal degradation of activated PERK; depletion of PDI reduces PERK activity and increases cellular vulnerability to ER stress–induced damage (13). A recent study further identified TRIM29 as a PERK-interacting post-translational regulator during cardiotropic virus infection. In cardiomyocytes, virus-induced TRIM29 promotes predominantly SUMO1-mediated PERK SUMOylation and maintains PERK stability, thereby enhancing PERK–ATF4–CHOP signaling, ER stress-associated apoptosis, and ROS responses (14). Although this mechanism was characterized in viral myocarditis, it reveals an additional layer of PERK regulation that may inform future studies of PERK-driven stress adaptation in cancer. In addition, mutant p53 interacts with PERK and the stress granule component G3BP1, thereby inhibiting stress granule assembly and increasing cellular sensitivity to ER stress–inducing agents (15). Collectively, these findings demonstrate that protein–protein interactions and post-translational modifications regulate PERK activation and stability in tumor cells and thereby shape cellular sensitivity to ER stress.

##### 2.1.1.2 Translational reprogramming and metabolic adaptation

PERK-mediated phosphorylation of eIF2α enables selective IRES-dependent translation of specific oncogenic transcripts, including HIF1α and c-Myc, thereby promoting metabolic reprogramming in pancreatic ductal adenocarcinoma. Through this mechanism, PERK signaling enhances the Warburg effect, enabling tumor cell proliferation and survival under oxygen–glucose deprivation conditions (16). In hepatocellular carcinoma, PERK activation mediated by PGRMC1 suppresses c-Myc translation in an ER stress–independent manner, resulting in tumor growth inhibition (17). These context-dependent outcomes demonstrate that PERK-controlled translational reprogramming can either promote or suppress tumor progression depending on molecular context.

##### 2.1.1.3 Epigenetic reprogramming and metastatic fitness

PERK signaling regulates epigenetic remodeling through ATF4-dependent induction of MAT2A, which increases intracellular S-adenosylmethionine levels and promotes H3K4me3 deposition at the PDGFB promoter. This epigenetic modification enhances survival of circulating tumor cell clusters in the bloodstream and facilitates metastatic dissemination (18). Thus, PERK–ATF4 signaling directly links ER stress to chromatin remodeling and metastatic competence.

##### 2.1.1.4 Lipid metabolism and immune microenvironment regulation

In IDH-mutant glioma, PERK activation promotes cholesterol efflux through regulation of lipid metabolic pathways, thereby modulating tumor cell invasiveness. This PERK-driven cholesterol export also contributes to M1-like macrophage polarization within the tumor microenvironment (19).

These findings indicate that PERK signaling coordinates lipid metabolism with immune contexture in specific oncogenic settings.

##### 2.1.1.5 Redox homeostasis and ferroptosis sensitivity

PERK signaling plays an important role in regulating redox balance and ferroptosis sensitivity in tumor cells. Loss of PERK reduces ATF4-dependent expression of the cystine transporter SLC7A11, thereby limiting antioxidant capacity and promoting lipid peroxidation and ferroptosis cell death in colorectal cancer cells (20). In pancreatic ductal adenocarcinoma, the scaffold protein cTRIP12 promotes ferroptosis resistance and immune evasion by interacting with PERK and facilitating OGT-dependent O-GlcNAcylation, which increases the expression of ferritin heavy chain and PD-L1 in tumor cells (21). Collectively, these findings indicate that PERK signaling coordinates redox homeostasis and ferroptosis sensitivity, thereby influencing tumor immune resistance.

##### 2.1.1.6 PERK–NRF2–MRP1 axis and multidrug resistance

Adaptation to ER stress can promote the acquisition of a multidrug-resistant (MDR) phenotype in tumor cells. Mechanistically, this is mediated by the PERK–NRF2–MRP1 signaling axis: PERK promotes NRF2-dependent transcription of the drug efflux transporter MRP1, thereby conferring resistance to both ER stress and chemotherapeutic agents. Disruption of the PERK–NRF2 pathway simultaneously restores sensitivity to ER stress and chemotherapy, indicating that this axis is essential for maintaining the multidrug-resistant phenotype (22). In addition, PERK can phosphorylate and stabilize NRF2 in KEAP1 wild-type tumor cells, protecting NRF2 from degradation and supporting cellular adaptation to stress conditions (23).

#### 2.1.2 Biological consequences of PERK activation in tumors

##### 2.1.2.1 Apoptosis and autophagy regulation

Activation of the PERK–ATF4–CHOP axis induces apoptosis and cell cycle arrest under severe ER stress. In hepatocellular carcinoma, ASS1 overexpression enhances PERK signaling, suppressing tumor growth and increasing chemosensitivity independently of p53 or arginine metabolism (24). In colorectal cancer, the proapoptotic fragment p20BAP31 localizes to the ER and hyperactivates PERK, promoting ROS accumulation that triggers both apoptosis and autophagy (12). p20BAP31-induced autophagy involves PI3K/AKT/mTOR inhibition and acts cooperatively with apoptosis to reduce tumor growth *in vivo* (25). These findings demonstrate that sustained PERK signaling can shift tumor cells from adaptive survival to programmed cell death.

##### 2.1.2.2 Immunogenic cell death and antitumor immunity

Gemcitabine induces immunogenic cell death in tumor cells via PERK activation, promoting calreticulin exposure and release of damage-associated molecular patterns (DAMPs), which stimulate dendritic cell maturation and CD8^+^ T cell-mediated antitumor responses (26). Ablation or pharmacological inhibition of PERK enhances antitumor immunity by inducing SEC61β-mediated paraptosis and increasing type I interferon signaling, while also reprogramming tumor-associated macrophages from the immunosuppressive M2 phenotype to the antitumor M1 phenotype, collectively suppressing tumor growth (27, 28). These findings highlight PERK as a central regulator that links tumor-intrinsic ER stress responses with immune activation, and suggest its potential as a therapeutic target to enhance immunogenic cell death and antitumor immunity.

##### 2.1.2.3 Tumor growth and invasion

Overexpression of PERK suppresses proliferation, migration, and epithelial–mesenchymal transition (EMT) in pancreatic ductal adenocarcinoma while promoting apoptosis (29). In hepatocellular carcinoma, PERK activation mediated by PGRMC1 inhibits tumor growth via suppression of c-Myc translation (17). Conversely, PERK-dependent translational control enhances tumor growth in pancreatic ductal adenocarcinoma through metabolic reprogramming (16). These findings collectively demonstrate that PERK signaling exerts context-dependent effects on tumor growth and invasiveness.

### 2.2 IRE1α signaling in RNA regulation and tumor progression

**Figure 2:**
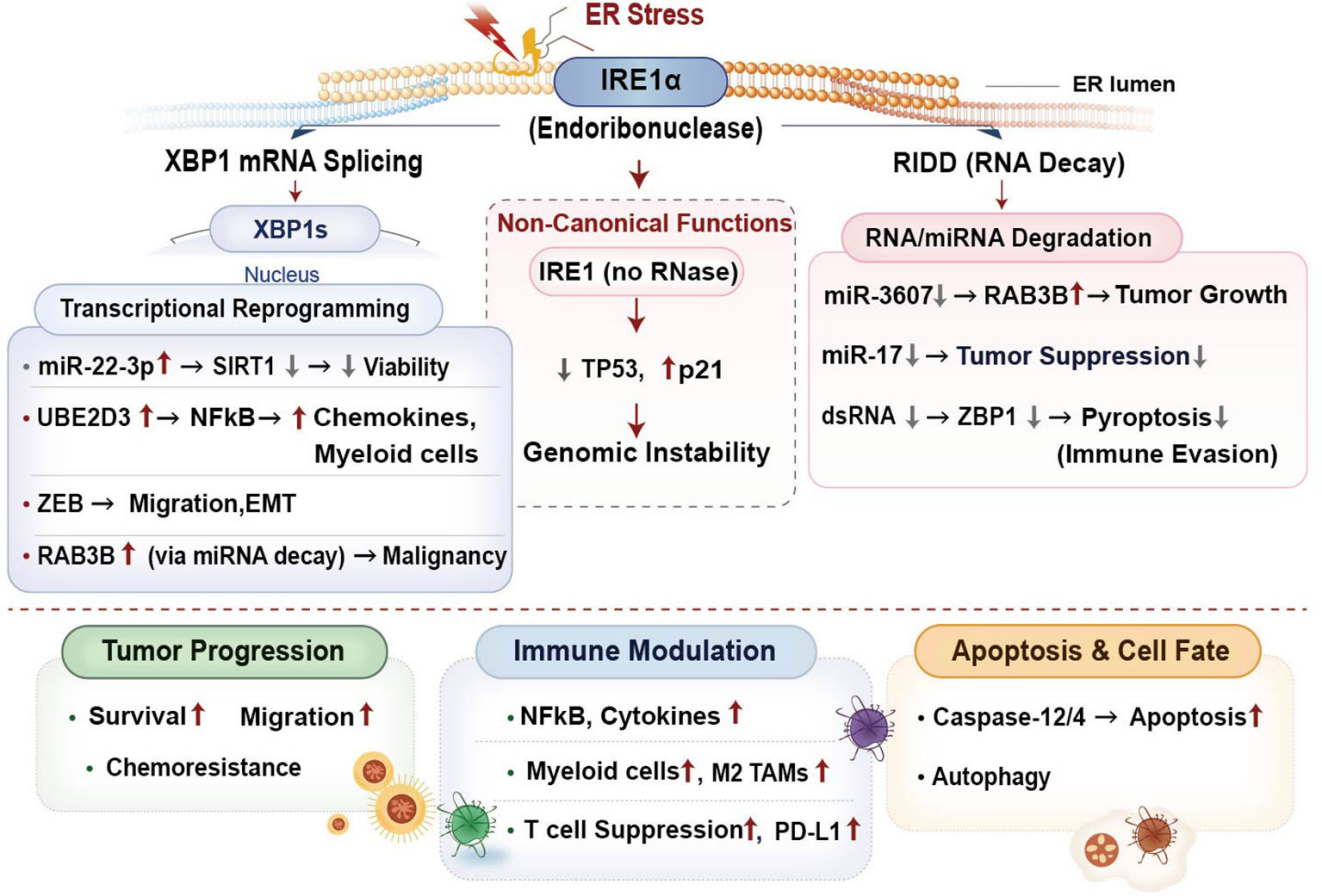
IRE1α signaling in RNA regulation and tumor progression. ER stress activates the endoribonuclease activity of IRE1α, leading to XBP1 mRNA splicing and RIDD. These pathways remodel transcriptional and post-transcriptional programs that influence tumor growth, immune modulation, apoptosis, and cell fate decisions. In addition, IRE1α can exert non-enzymatic scaffolding functions that contribute to genomic instability and tumor progression.

The IRE1α pathway integrates ER stress into dual downstream outputs mediated by its endoribonuclease activity: unconventional splicing of XBP1 mRNA to generate the transcription factor XBP1s, and RIDD of selected RNAs. These processes can exert context-dependent and even antagonistic effects on tumor progression (**Figure 2**) (30).

#### 2.2.1 Molecular regulatory mechanisms of IRE1α signaling

##### 2.2.1.1 XBP1s-mediated transcriptional reprogramming

IRE1α endoribonuclease activation splices XBP1 mRNA to produce XBP1s, which transcriptionally regulates genes involved in survival and stress adaptation. In leukemic cells, XBP1s upregulates miR-22-3p, which targets SIRT1 and reduces cell viability while enhancing chemosensitivity (31). In glioblastoma, IRE1 signaling through XBP1s regulates UBE2D3 expression, which activates NFκB and promotes chemokine production and myeloid infiltration (32). In luminal breast cancer, IRE1–XBP1 signaling induces degradation of tumor suppressor miRNAs via RIDD, leading to elevation of RAB3B and enhanced malignancy (33). In liver and breast cancer cells, palmitate activates the IRE1–XBP1 pathway to induce ZEB transcription, repress desmoplakin, and promote migration (34). In germinal-center B-cell-like diffuse large B-cell lymphoma, reduced IRE1 expression due to EZH2-mediated H3K27 trimethylation prevents XBP1 activation, while pharmacological inhibition of EZH2 restores IRE1 signaling and suppresses tumor growth (35). These data indicate that XBP1s-driven transcription can either promote or restrain tumor growth depending on epigenetic and cellular context.

##### 2.2.1.2 RIDD-mediated RNA decay

Beyond XBP1 splicing, IRE1α mediates degradation of specific RNAs through RIDD (30, 33). IRE1-dependent degradation of tumor suppressor miR-3607 results in upregulation of RAB3B and promotes malignancy in breast cancer (33). In triple-negative breast cancer, IRE1 RNase silences taxane-induced double-stranded RNA via RIDD, thereby preventing ZBP1 sensing and suppressing NLRP3–GSDMD-mediated pyroptosis (36). In glioblastoma, IRE1‐downstream signals play antagonistic roles in cancer development, where XBP1s provides pro‐tumoral signals, whereas RIDD of mRNA and miR17 rather elicits anti‐tumoral features (30). Thus, RIDD activity can either support immune escape or exert tumor-restrictive effects depending on substrate selection and tumor context.

##### 2.2.1.3 Non-canonical and Non-enzymatic functions of IRE1

Some cancer cell lines require IRE1 protein expression independently of its RNase activity. IRE1 knockdown, but not enzymatic inhibition, attenuates cell cycle progression and tumor growth while activating TP53 and CDKN1A/p21 and increasing chromosomal instability (37). These findings indicate that IRE1 can support tumor growth through non-enzymatic scaffolding functions.

#### 2.2.2 IRE1α signaling in tumor–immune regulation

IRE1α signaling modulates immune recognition and inflammatory outputs within the tumor microenvironment. In triple-negative breast cancer, inhibition of IRE1 enhances taxane-induced dsRNA accumulation and promotes ZBP1-dependent pyroptosis, thereby restoring innate immune recognition of immunologically cold tumors (36). Consistently, inhibition of IRE1 RNase attenuates protumorigenic cytokine signaling and synergizes with paclitaxel to enhance antitumor efficacy (38). In glioblastoma, IRE1/UBE2D3 signaling activates NFκB and drives chemokine production and myeloid cell infiltration (32). In high-grade serous ovarian cancer, neutrophil-intrinsic IRE1α suppresses T cell antitumor responses and promotes early adaptive immune escape (39). However, deletion of IRE1 RNase and XBP1s in dendritic cells does not universally alter tumor growth or T cell effector responses in melanoma and colon cancer models, arguing against a generalized protumorigenic role in all DC subsets (40). Thus, the immunological impact of IRE1 signaling is highly cell-type dependent.

#### 2.2.3 IRE1α in apoptosis and cell fate determination

Activation of IRE1α can directly induce cell death pathways under specific contexts. Pinocembrin induces ER stress via IRE1α/XBP1 signaling and triggers caspase-12/-4-mediated apoptosis in melanoma cells (41). GPx8 regulates apoptosis and autophagy through the IRE1/JNK pathway in esophageal squamous cell carcinoma (42). IRE1 signaling tightly controls CD95-dependent cell death via coordinated actions of RIDD and XBP1s (43). In Drosophila and mammalian models, IRE1 activity regulates tumor-suppressive cell competition by modulating apoptosis and autophagy, and relative IRE1 activity determines loser cell fate (44). These findings establish IRE1 as a critical regulator of stress-induced apoptosis and competitive elimination of transformed cells.

### 2.3 ATF6 signaling in proteostasis control and tumor plasticity

**Figure 3:**
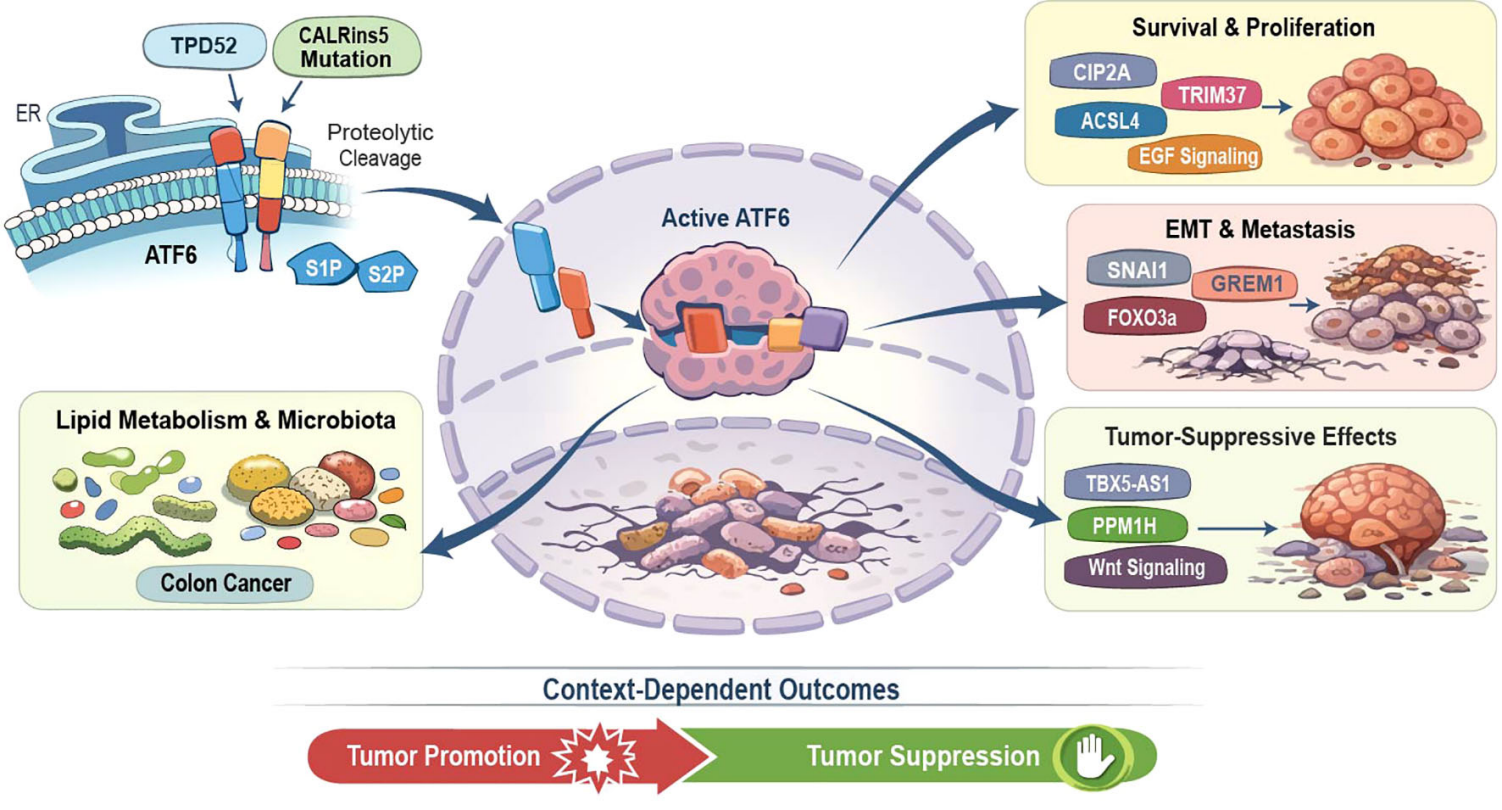
ATF6 signaling in proteostasis control and tumor plasticity. Under ER stress, ATF6 undergoes proteolytic cleavage by S1P/S2P in the Golgi apparatus to generate the active transcription factor that translocates to the nucleus and regulates gene expression. ATF6 signaling influences multiple tumor-related processes, including cell proliferation, EMT and metastasis, metabolic regulation, and tumor-suppressive responses.

ATF6 is a membrane-bound ER stress sensor that undergoes S1P/S2P-mediated proteolytic cleavage to generate an active transcription factor that reprograms proteostasis, metabolism, and cell fate decisions. Its activity in cancer is highly context-dependent, functioning either as a tumor promoter or tumor suppressor depending on upstream regulatory inputs and downstream transcriptional targets (**Figure 3**) (45, 46).

#### 2.3.1 Upstream regulation and activation of ATF6

Tumor protein D52 (TPD52) promotes S2P-mediated cleavage of ATF6 and integrates ER stress with the chaperone machinery. Depletion of TPD52 attenuates ER stress signaling and facilitates tumorigenesis in a subset of human carcinomas (47). CALRins5 mutations activate ATF6 due to loss of CALR chaperone function, and CALR-mutant cells partially depend on ATF6 for cytokine-independent growth. ATF6 directly induces expression of BCL-XL in CALR-mutant cells, promoting survival (48). These findings indicate that ATF6 activation can be driven by alterations in ER chaperone systems and may create survival dependencies in genetically defined tumors.

#### 2.3.2 ATF6-driven transcriptional programs in tumor cells

##### 2.3.2.1 Survival and proliferation signaling

ATF6 directly binds to the CIP2A promoter and induces its expression, thereby supporting colon cancer cell survival (49). In cervical cancer, ATF6 transcriptionally upregulates TRIM37, which promotes ubiquitination and degradation of ACSL4, leading to ferroptosis suppression and enhanced tumor progression (50). In prostate cancer, ATF6 depletion reduces Golgi fragmentation and disrupts glycosylation processes that regulate integrin αv and Gal-3 trafficking, and combined ATF6 depletion with autophagy inhibition suppresses tumor growth and metastasis (51). In non-small cell lung cancer, the ATF6–EGF signaling axis in slow-cycling cancer cells promotes the angiogenic switch following chemotherapy, driving recurrence through reactivation of proliferative tumor cells (52). Together, these findings establish ATF6 as a regulator of survival signaling, membrane trafficking, and post-therapy tumor reactivation.

##### 2.3.2.2 EMT and metastatic reprogramming

ATF6 forms a self-amplifying loop with SNAI1 that drives EMT-like changes and sustains UPR-dependent EMT programs (53). In colorectal cancer, GREM1 enhances invasion and EMT by upregulating ATF6 expression and modulating UPR signaling through PI3K/AKT/mTOR activation (54). ATF6α reduces ΔNp63α expression through GRP78-mediated activation of AKT1, leading to FOXO3a degradation and promotion of metastasis (55). Cancer cell-derived ATF6 can disrupt endothelial tight junction proteins ZO-1 and VE-cadherin, increasing vascular permeability and facilitating brain metastasis (56). Susceptibility to inflammatory bowel disease enhances invasive carcinomas in ATF6-driven colon cancer models, indicating that chronic inflammatory stress can cooperate with ATF6 signaling to promote tumor progression (57). These studies demonstrate that ATF6 reprograms epithelial plasticity and microenvironmental barriers to facilitate metastatic dissemination.

##### 2.3.2.3 Lipid metabolism and microbiota-dependent tumorigenesis

ATF6 activation enriches long-chain fatty acids in the colonic epithelium, reshapes gut microbiota composition, and promotes colorectal tumorigenesis (58). In nATF6IEC mice, sustained intestinal ATF6 activation drives dysbiosis and microbiota-dependent tumor formation, and high ATF6 expression correlates with reduced disease-free survival in patients with colorectal cancer (59). These findings link ATF6-driven ER stress responses to lipid metabolism and microbiota-mediated tumor progression.

##### 2.3.2.4 Context-dependent effects on tumor growth

Activation of ATF6 reduces proliferation and intestinal epithelial stemness in certain models, indicating potential tumor-suppressive functions (46). TBX5-AS1 activates ER stress signaling through the miR-494-3p/ATF6 axis and suppresses lung cancer growth and stemness (45). In hepatocellular carcinoma, ATF6 suppresses PPM1H expression, and PPM1H inhibits tumor progression by dephosphorylating RPS6KB1, suggesting complex regulatory interactions between ATF6 signaling and growth control pathways (60). In hepatocellular carcinoma, CYP2E1 induces ER stress and upregulates ATF6, GRP78, and CHOP, while modulating apoptotic regulators including BCL2 and caspase-3 (61). ATF6 intervention reduces colorectal cancer viability by interrupting dysregulated Wnt signaling (62). These findings highlight the context-dependent effects of ATF6 signaling on tumor growth.

### 2.4 Crosstalk and adaptive compensation among UPR pathways

The three canonical branches of the UPR—PERK, IRE1α, and ATF6—do not function as isolated signaling modules, but instead operate as an interconnected adaptive network that dynamically coordinates stress adaptation, proteostasis, and cell fate determination. Increasing evidence indicates that extensive crosstalk and compensatory signaling exist among these pathways, enabling tumor cells to maintain ER homeostasis under persistent stress conditions while simultaneously limiting the efficacy of single-pathway therapeutic interventions.

Reciprocal regulation between PERK and IRE1α represents a central adaptive feature of the UPR network, but operates through distinct mechanistic layers and stress contexts. In pancreatic neuroendocrine tumors, inhibition of either PERK or IRE1α induces compensatory hyperactivation of the reciprocal branch, which ultimately overwhelms ER homeostasis and shifts signaling from adaptation to apoptosis, thereby suppressing tumor growth (63). A similar reciprocal imbalance has been observed in vascular tumors, where elevated PERK activity is associated with reduced IRE1α signaling and diminished XBP1 splicing, whereas PERK downregulation enhances IRE1α–XBP1 activation under ER stress (64). These findings support a “stress-switch” model in which UPR branches functionally compensate within a limited range until a threshold is exceeded, triggering cell death. Importantly, these apparently divergent observations likely reflect context-dependent regulation of UPR signaling, wherein acute stress favors reciprocal antagonism between PERK and IRE1α, whereas chronic ER stress promotes interdependent maintenance of basal UPR activity. Consistent with this distinction, chronic-stress models demonstrate a maintenance-level interdependence between PERK and IRE1α signaling. Inhibition of PERK reduces IRE1 expression, whereas suppression of IRE1 signaling decreases PERK protein abundance and downstream signaling activity through disruption of the IRE1–XBP1 axis (65). Mechanistically, IRE1 RNase activity contributes to sustaining PERK expression via XBP1s-dependent signaling, indicating that basal UPR activity requires coordinated transcriptional and post-transcriptional support between branches rather than simple compensatory overactivation (65). Together, these studies indicate that UPR crosstalk operates at multiple regulatory layers, including both adaptive compensation and pathway interdependence, which together determine tumor cell fate under ER stress.

Crosstalk among UPR branches is further supported by studies involving ATF6 signaling. In colorectal cancer cells, enforced activation of either XBP1s or ATF6 reduced stemness and proliferation through cross-activation of the PERK–eIF2α pathway. Mechanistically, XBP1-induced suppression of proliferation could be rescued by constitutive expression of the eIF2α phosphatase GADD34, indicating that the PERK–eIF2α axis mediates, at least in part, the growth-suppressive effects triggered by XBP1s activation (46). Moreover, ATF6α deficiency induced sustained activation of both IRE1 and PERK signaling pathways, indicating that loss of ATF6α can be compensated by enhanced activity of the remaining UPR branches in HCT116 colorectal cancer cells. Interestingly, this compensatory mechanism differed from that observed in mouse embryonic fibroblasts, where ATF6β compensated for ATF6α deficiency. Importantly, combined disruption of IRE1 and ATF6α signaling significantly impaired tumor growth compared with single-pathway inhibition alone (66). These findings demonstrate that tumor cells can employ distinct context-specific compensation strategies to preserve ER homeostasis and maintain survival under therapeutic pressure.

Overall, emerging evidence indicates that UPR signaling functions as a highly integrated and plastic stress-adaptive network rather than a collection of independent pathways. Dynamic crosstalk and compensatory activation among PERK, IRE1α, and ATF6 enable tumor cells to maintain proteostasis and survive fluctuating microenvironmental stress, but simultaneously limit the long-term efficacy of single-pathway therapeutic strategies. These observations suggest that UPR crosstalk not only sustains adaptive signaling, but also helps determine the threshold at which persistent ER stress becomes therapeutically exploitable and shifts toward tumor cell death (67, 68). Thus, clarifying how UPR branches compensate for, depend on, or antagonize one another under therapeutic pressure may provide a mechanistic basis for designing combination strategies that overcome adaptive compensation in cancer.

## 3 Small-molecule inhibitors of the UPR pathways

**Figure 4:**
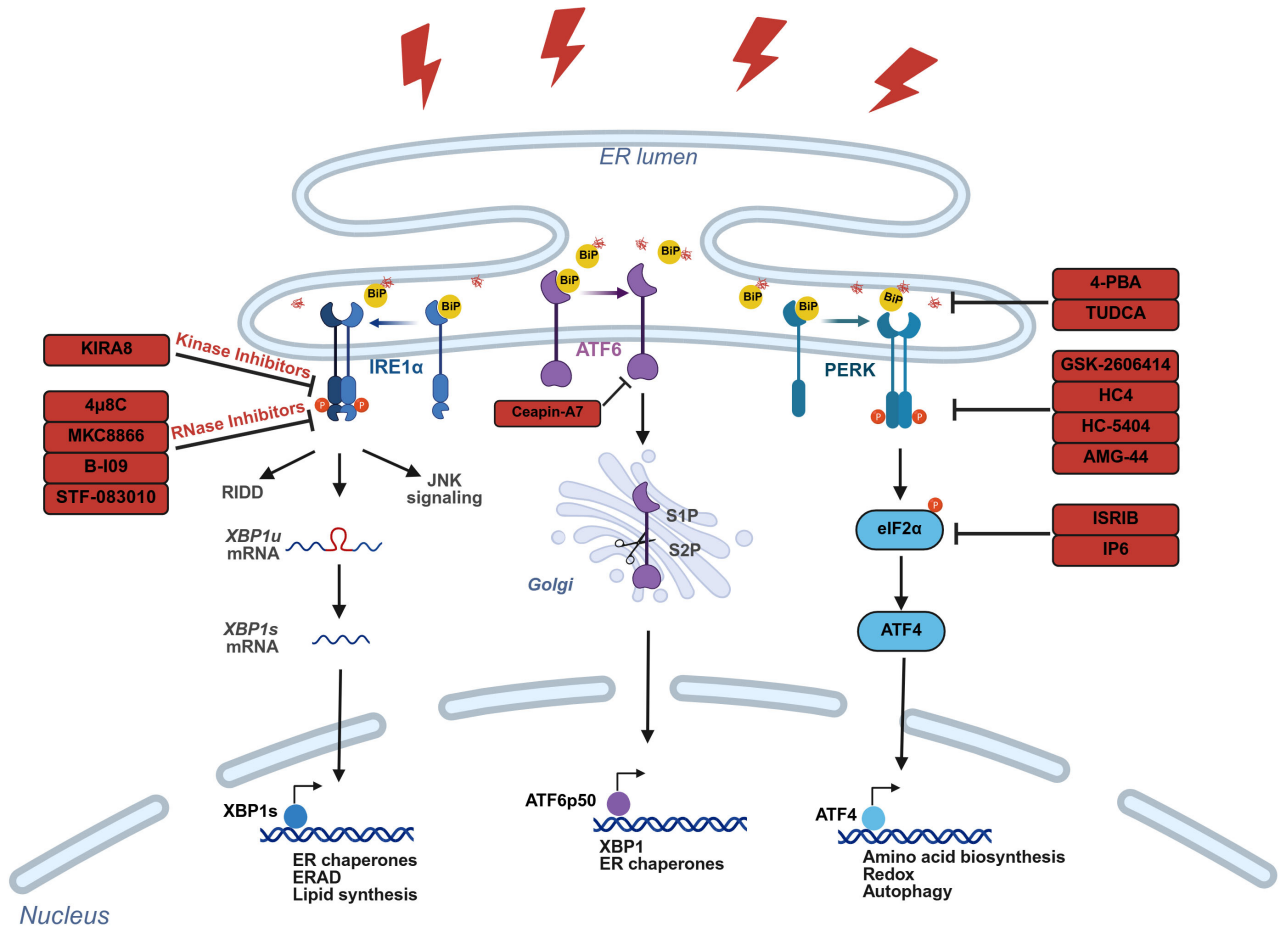
Small-molecule inhibitors of the UPR pathways. The UPR is activated by ER stress through three primary pathways: IRE1α, PERK, and ATF6. IRE1α splices XBP1 mRNA, mediates RIDD, and activates JNK signaling; its inhibitors include RNase inhibitors (e.g., 4μ8C, MKC8866) and kinase inhibitors (e.g., KIRA8). PERK phosphorylates eIF2α, attenuates protein translation and enhances ATF4 expression, which regulates genes involved in redox balance, autophagy, and amino acid metabolism; key inhibitors include GSK2606414 and ISRIB. ATF6 traffics to the Golgi apparatus and undergoes S1P/S2P-mediated cleavage to generate the active ATF6p50 transcription factor, which induces ER chaperone and ERAD gene expression; Ceapin-A7 selectively inhibits ATF6α activation by blocking its stress-induced Golgi translocation. Chemical chaperones (e.g., 4-PBA, TUDCA) reduce ER stress by enhancing protein folding capacity, although they may exhibit dual roles in tumor suppression and promotion depending on the context. Created in BioRender. Lin, M. (2026) https://BioRender.com/nymyq1d.

The UPR is essential for tumor cell survival and adaptation under conditions of ER stress. When tumor cells experience ER dysfunction, UPR signaling pathways are activated to mitigate stress and maintain cellular homeostasis. UPR signaling not only drives malignant progression but also confers resistance to therapeutic interventions. Consequently, targeting key components of the UPR has emerged as a promising strategy in cancer therapy. Selective inhibition of UPR pathways can either induce tumor cell death or restore sensitivity to existing treatments. Among the three primary UPR branches, significant progress has been made in developing inhibitors targeting the IRE1α and PERK pathways, whereas research on ATF6 pathway inhibitors remains in its early stages. This section summarizes advances in targeting the IRE1α, PERK, and ATF6 pathways, together with chemical chaperone–based ER stress modulation, for cancer treatment (**Figure 4**).

### 3.1 IRE1α pathway inhibitors

IRE1α, a type I transmembrane protein located in the ER, serves as one of the primary sensors of ER stress. It helps maintain intracellular protein homeostasis primarily through the activation of XBP1s. Notably, IRE1α exhibits dual enzymatic activity, functioning as both a kinase and an endoribonuclease (RNase), which enables it to regulate various physiological processes such as mRNA and protein degradation, inflammation, immune responses, cell proliferation, and apoptosis (69). IRE1α inhibitors, which target its dual enzymatic activities, are categorized into kinase inhibitors and RNase inhibitors based on their distinct mechanisms of action. Kinase inhibitors such as KIRA8 selectively suppress IRE1α’s kinase activity, blocking autophosphorylation and downstream signaling pathways. RNase inhibitors such as 4μ8C, MKC8866, B-I09, and STF-083010 directly suppress the endoribonuclease activity of IRE1α, thereby preventing the unconventional splicing of XBP1 mRNA and subsequent production of the transcriptionally active XBP1s isoform (3, 70, 71). These inhibitors collectively modulate IRE1α-driven processes, including UPR activation, inflammatory signaling, and cell fate decisions (**Table 1**), offering therapeutic potential in cancer (3).

**Table 1:** IRE1α inhibitors in cancer therapy.

Inhibitor	Cancer type	Effects	Mechanisms	Advantages	Limitations	Clinical status
IRE1α Kinase Inhibitors						
KIRA8	MM	Induces apoptosis; suppresses tumor growth; enhances the efficacy of bortezomib, asciminib, and lenalidomide (72–74)	↑ CHOP expression; ↓ XBP1 splicing; ↓ immunoglobulin light-chain and cytokine/chemokine secretion	Selective IRE1α kinase-domain inhibitor with allosteric RNase-attenuating activity; demonstrates activity across multiple preclinical tumor models and may enhance proteotoxic stress, radiotherapy, anti-myeloma therapy, and antiangiogenic combinations.	Pharmacokinetic and toxicity profiles remain insufficiently defined; efficacy may depend on tumor ER-stress/IRE1α dependency and kinase -RNase coupling, and clinical translation data remain limited.	Preclinical
KIRA8	PDAC	Enhances radiotherapy-induced apoptosis (75)	↓IRE1α kinase activity			
KIRA8	CRC	Suppresses proliferation (76)	↓JNK phosphorylation			
KIRA8	PanNET	Suppresses tumor growth (63)	induces apoptosis			
KIRA8	TNBC	Inhibits tumor growth; synergizes with anti-VEGF therapy (77)	Modulates tumor microenvironment			
IRE1α RNase Inhibitors						
4μ8C	HCC	Enhances doxorubicin efficacy; reduces fibrosis (78)	↓ER stress/lipid metabolism;	Widely used IRE1α RNase inhibitor; useful for blocking XBP1 splicing/RIDD and testing chemotherapy-sensitizing or tumor–stroma effects	Mainly a preclinical tool compound; context-dependent effects due to variable roles of XBP1s and RIDD	Preclinical
4μ8C	BC	Suppresses growth and migration (33)	Blocks RIDD-mediated miRNA degradation			
4μ8C	CRC	Suppresses growth and migration, stemness; enhances 5-FU sensitivity (79–81)	↓ β-catenin, ↓ ABC transporters (ABCB1/MDR1)			
4μ8C	Choriocarcinoma	Attenuates GRA15II-induced apoptosis (82)	↓XBP1s/CHOP/TRAF			
4μ8C	Lymphoma	inhibits EBV-driven lymphomagenesis; Suppresses tumor growth, enhances sensitivity of AZD2461 (83, 84)	Inhibits IRE1α; disrupts HR repair/↓ c-Myc			
MKC8866	PCa	Suppresses growth; enhances anti-PD-1 efficacy (85, 86)	↓ M2 macrophages, ↑ IFN-γ response	Most clinically advanced IRE1α RNase inhibitor; may enhance chemotherapy, targeted therapy, and immunotherapy through inhibition of XBP1 splicing/RIDD and immune remodeling	Optimal predictive biomarkers, responsive tumor types, and combination schedules remain to be defined	Phase I/II; NCT03950570
MKC8866	HCC	Inhibits Wnt/LEF1-active tumors (87)	Blocks XBP1 splicing			
MKC8866	GBM	Synergizes with Stupp protocol (TMZ + radiotherapy) (88)	Inhibits IRE1α/XBP1 axis			
MKC8866	TNBC	Enhances paclitaxel efficacy (38)	Attenuates autocrine and paracrine signals of protumorigenic factors			
MKC8866	OC	Synergizes with AZD1775 (WEE1 inhibitor) (89)	Targets IRE1α/XBP1 pathway in TP53-mutant cells			
MKC8866	RMS	Reduces proliferation; induces senescence (90)				
MKC8866	Ph+ ALL	Synergizes with nilotinib (91)				
MKC8866	KRAS-mutant LC/PC	Synergizes with sotorasib/trametinib (92)				
MKC8866	TNBC	enhances chemotherapy and immunotherapy efficacy (36)	↑ dsRNA accumulation, activates NLRP3-GSDMD pathway			
B-I09	OC	Suppresses growth; synergizes with anti-PD-1 and HDAC6 inhibitor (93, 94)	↓CARM1-dependent IRE1α/XBP1 signaling	IRE1α RNase inhibitor with promising activity in ovarian cancer and hematologic malignancies; may support immunotherapy or targeted-combination strategies	Tumor subtype selection and clinical feasibility remain unclear	Preclinical
B-I09	CLL	Inhibits leukemia progression; Synergizes with BTK inhibitor (95)				
STF-083010	MM	Suppresses tumor growth; synergizes with ixazomib (96, 97)	Induces cell cycle arrest	Classical IRE1α RNase inhibitor widely used to block XBP1 splicing in experimental models; useful for mechanistic and combination studies	Limited drug-like properties and context-dependent efficacy	Preclinical
STF-083010	CRC	Suppresses cancer cell proliferation (76)	Inhibits IRE1α RNase activity, blocks RIDD			
STF-083010	TNBC	Enhances pro-apoptotic activity; synergizes with BIKDD (98)	Stabilizes BIKDD, prevents its degradation			
STF-083010	NB	Blocks KFL induced differentiation (99)				

BC, breast cancer; CLL, chronic lymphocytic leukemia; CRC, colorectal cancer; GBM, glioblastoma; HCC, hepatocellular carcinoma; LC, lung cancer; MM, multiple myeloma; NB, neuroblastoma; OC, ovarian cancer; PanNET, pancreatic neuroendocrine tumor; PC, pancreatic cancer; PCa, prostate cancer; PDAC, pancreatic ductal adenocarcinoma; Ph+ ALL, Philadelphia chromosome-positive acute lymphoblastic leukemia; RMS, rhabdomyosarcoma; TNBC, triple-negative breast cancer. ↑ indicates increased or upregulated; ↓ indicates decreased or downregulated.

#### 3.1.1 IRE1α kinase inhibitors

The kinase activity of IRE1α is crucial for tumor cell survival and adaptation to ER stress. Inhibiting this activity disrupts these adaptive mechanisms, leading to tumor cell death. Additionally, conformational changes in the IRE1α kinase domain regulate its RNase function, unveiling an allosteric mechanism within the UPR (100). This insight provides a mechanistic basis for developing IRE1α-targeted strategies in cancer. Recently, IRE1α kinase inhibitors have attracted growing attention as a promising anticancer strategy, with studies highlighting their potential in cancer therapy. Several IRE1α kinase-domain inhibitors have been developed, among which KIRA8, also known as Compound 18, has been extensively evaluated in preclinical cancer models.

KIRA8, also known as Compound 18, is a selective kinase-inhibiting RNase attenuator that binds the ATP pocket of the IRE1α kinase domain and allosterically attenuates its RNase activity. In multiple myeloma, KIRA8 induces apoptosis and enhances the efficacy of bortezomib or asciminib (72, 73). It also suppresses tumor growth, reduces the secretion of immunoglobulin light chains and tumor-promoting cytokines and chemokines, and enhances the antimyeloma efficacy of bortezomib and lenalidomide (74). In radiation-resistant pancreatic ductal adenocarcinoma, KIRA8 potentiates radiotherapy-induced apoptosis (75), whereas in colorectal cancer, it suppresses tumor growth by inhibiting IRE1α signaling and JNK phosphorylation (76). KIRA8 also inhibits pancreatic neuroendocrine tumor growth by inducing apoptosis (63). In triple-negative breast cancer, it suppresses tumor and patient-derived xenograft growth and enhances the efficacy of anti-VEGF-A therapy by remodeling the tumor microenvironment, including increasing pericyte abundance and reducing cancer-associated fibroblasts and myeloid-derived suppressor cells (77). Collectively, these findings support further preclinical evaluation of KIRA8 in IRE1α-dependent tumors and rational combination strategies.

#### 3.1.2 IRE1α RNase inhibitors

The RNase activity of IRE1α regulates mRNA splicing and degradation, primarily by activating XBP1s and mediating regulated RIDD, thereby influencing cell fate. Additionally, IRE1α RNase is essential for maintaining mRNA stability, particularly in DNA damage response pathways, modulating DNA repair, cell cycle arrest, and apoptosis (101). Notably, Myc overexpression induces ER stress by activating the IRE1α/XBP1 signaling pathway. This activation depends on the RNase function of IRE1α, which regulates lipid homeostasis to support the survival of Myc-transformed cancer cells (102). As a key regulator of cellular stress responses in both DNA damage repair and oncogenic transformation, IRE1α RNase plays a crucial role in tumor cell adaptation and survival. Therefore, IRE1α RNase inhibitors represent a promising therapeutic strategy with significant potential in cancer treatment. Several compounds have been developed to target IRE1α RNase activity, including 4μ8C, MKC8866, B-I09, and STF-083010. These inhibitors directly suppress the endoribonuclease activity of IRE1α, thereby preventing unconventional splicing of XBP1 mRNA and subsequent production of the transcriptionally active XBP1s isoform.

4μ8C demonstrates context-dependent dual functionality in cancer biology, primarily acting as a tumor suppressor through IRE1α RNase inhibition while occasionally exhibiting protumor effects. In cellular models, 4μ8C inhibits breast cancer proliferation and invasion by preventing RIDD-mediated degradation of tumor-suppressive miRNAs (33), suppresses EBV-driven B-cell immortalization and PTLD-like lymphoma survival (83), and sensitizes Burkitt lymphoma cells to PARP inhibition by disrupting homologous recombination repair and downregulating c-Myc (84). Context-dependent effects have also been observed: 4μ8C attenuates GRA15II-induced apoptosis in choriocarcinoma cells (82), whereas 4μ8C combined with 2-DG suppresses microcystin-LR-induced malignant phenotypes and M2 macrophage polarization in colorectal cancer (103). In animal models, 4μ8C shows antitumor efficacy: it enhances doxorubicin cytotoxicity in hepatocellular carcinoma (78), and suppresses proliferation and migration in hepatic stellate cells (104). In colorectal cancer, it inhibits tumor growth and stem cell activity (79), and reduces the expression of drug resistance-associated ABC transporters, thereby resensitizing resistant cells to 5-FU (80, 81). Collectively, 4μ8C suppresses IRE1α endoribonuclease activity, reduces ER stress, inhibits tumor growth, and enhances the efficacy of chemotherapeutic agents.

MKC8866, also known clinically as ORIN1001, is a selective inhibitor of the endoribonuclease activity of IRE1α and represents one of the most advanced UPR-targeting agents undergoing clinical translation. Extensive preclinical studies have demonstrated its broad-spectrum antitumor activity across multiple malignancies through distinct mechanisms. In prostate cancer, it synergizes with enzalutamide, abiraterone, and cabazitaxel while reprogramming immunosuppressive microenvironments by reducing tumor-associated macrophages and enhancing interferon responses, thereby potentiating anti-PD-1 immunotherapy (85, 86). Additionally, MKC8866 suppresses hepatocellular carcinoma growth (87), and enhances the efficacy of the Stupp protocol in glioblastoma (88). MKC8866 also enhances the antitumor effects of paclitaxel in triple-negative breast cancer (38) and synergizes with WEE1 inhibition (AZD1775) in mtTP53-mutant ovarian cancer (89). Its combination with TKIs (imatinib and nilotinib) improves outcomes in BCR-ABL1-positive leukemia (91). At the cellular level, MKC8866 reduces cell proliferation and clonogenicity, inducing senescence in rhabdomyosarcoma (90). In KRAS-mutant patient-derived xenograft models of lung and pancreatic cancers, MKC8866 combined with the KRAS inhibitor sotorasib or the MEK inhibitor trametinib significantly enhances therapeutic outcomes (92). Mechanistically, MKC8866 also enhances immunogenic cell death by disrupting the IRE1α-RIDD axis. This inhibition prevents taxane-induced dsRNA degradation, leading to NLRP3-GSDMD-mediated pyroptosis via accumulated dsRNA. This mechanism converts immunologically inert tumors into immunogenic hotspots, particularly overcoming immunosuppressive features in PD-L1-negative, TP53-mutant triple-negative breast cancer models (36). Importantly, MKC8866 has entered early-phase clinical development to evaluate its pharmacokinetics, safety profile, and therapeutic potential in patients with advanced solid tumors (70). Collectively, MKC8866 represents one of the most promising translational examples of targeting the IRE1α-XBP1 signaling pathway. However, further clinical studies are required to determine optimal combination strategies, predictive biomarkers, and patient populations that may benefit most from IRE1α inhibition.

B-I09 has shown preclinical efficacy in several tumor models. In ovarian cancer, B-I09 suppresses tumor growth via CARM1-dependent IRE1α/XBP1 blockade, and synergizes with anti-PD-1 antibodies (93) or HDAC6 inhibitors, particularly in ARID1A-deficient subtypes (94). Additionally, B-I09 monotherapy inhibits B-cell chronic lymphocytic leukemia progression in mice without systemic toxicity and shows combinatorial synergy with ibrutinib in B-cell malignancy models (95). By targeting the IRE1α-XBP1 pathway, B-I09 demonstrates remarkable therapeutic efficacy in ovarian cancer and hematologic malignancies. Its combined use with other agents further enhances its antitumor activity, highlighting its substantial potential for clinical development.

STF-083010 exhibits context-dependent effects, showing antitumor activity in several models while attenuating treatment-induced differentiation or cytotoxicity in selected settings. In multiple myeloma models, STF-083010 demonstrates significant antitumor activity (96) and synergizes with ixazomib to induce cell cycle arrest and enhance cytotoxicity (97). Additionally, STF-083010 stabilizes BIKDD to improve antitumor efficacy without observed toxicity in triple-negative breast cancer models (98), and suppresses colorectal cancer progression (76). Paradoxically, STF-083010 markedly suppresses quercetin (KFL)-induced neuroblastoma differentiation (99) and diminishes therapeutic synergy between protein synthesis inhibitors and UPR inducers (105), underscoring its functional dichotomy across tumor types.

From a translational perspective, most IRE1α inhibitors discussed above, including 4μ8C, STF-083010, KIRA8, and B-I09, remain preclinical tool compounds, whereas MKC8866 represents the most clinically advanced IRE1α RNase inhibitor. MKC8866 was evaluated in a completed Phase I/II trial in patients with advanced solid tumors or relapsed/refractory metastatic breast cancer (NCT03950570). This makes IRE1α RNase inhibition the most clinically advanced branch-specific UPR-targeted strategy to date. Nevertheless, its further clinical development will require clearer pharmacodynamic monitoring of IRE1α RNase inhibition, including XBP1 splicing and RIDD activity, as well as biomarker-guided patient selection and rational combination strategies tailored to tumor contexts with strong IRE1α dependence.

### 3.2 PERK pathway inhibitors

The activation of the UPR is critical for cancer cell survival under stress conditions. Within the UPR, the PERK pathway enhances cellular respiratory capacity under stress conditions by regulating mitochondrial protein import and cristae formation (106). Additionally, PERK influences antitumor immune responses by modulating immune escape mechanisms. Specifically, PERK downregulation activates robust antitumor immune responses, promotes immunogenic cell death, and enhances antitumor efficacy (27). Given its pivotal role in both cancer cell survival and immune modulation, targeting the PERK pathway offers a promising therapeutic strategy (**Table 2**).

**Table 2:** PERK inhibitors in cancer therapy.

Inhibitor	Cancer type	Effects	Mechanisms	Advantages	Limitations	Clinical status
GSK2606414	BC	Overcomes resistance in FOXO3-dependent resistant cells (107)	↓PERK activity	Potent and widely used PERK inhibitor; useful for reversing chemoresistance and dissecting PERK-dependent stress adaptation across multiple tumor models	Systemic PERK inhibition may cause proteostasis-related toxicity; may antagonize ER stress-induced tumor cell death in some contexts	Preclinical
GSK2606414	OC	Potentiates metformin-induced apoptosis; mitigates EVO-induced apoptosis (108, 109)	autophagy and the PERK/eIF2α pathway			
GSK2606414	PDAC	Suppresses CAF transformation and tumor angiogenesis; enhances gemcitabine efficacy and reverses RUNX1-mediated resistance (16, 110, 111)	↓PERK/eIF2α phosphorylation; ↓ERK1/2 phosphorylation			
GSK2606414	CRC	Overcomes resistance; synergizes with ERO1L inhibition; reverses WZ35-induced apoptosis (112–114)	↓PERK activity			
GSK2606414	HCC	Enhances folate deficiency-induced apoptosis (115)	disruption of the PERK-ATF4-LAMP3 pathway			
GSK2606414	GBM	Enhances temozolomide + simvastatin efficacy; prevents GBM recurrence (116, 117)	↓PERK-driven autophagy; eliminates senescent cells			
GSK2606414	medulloblastoma	suppresses tumor cell migration and invasion (118)	↓PERK activation			
GSK2606414	Leukemia	Overcomes resistance to G9a inhibitors; synergizes with digoxin (119, 120)	↓ PERK/NRF2 pathway			
GSK2606414	OS	Blocks CYT997 (lexibulin)-induced apoptosis and autophagy (121)	↓ ROS-dependent ER stress activation via PERK suppression			
ISRIB	TNBC	Enhances doxorubicin sensitivity; synergizes with ERO1 deficiency; suppresses ETHE1-driven metastasis; enhances nanodrug-photothermal therapy (122–125)	Modulates stress-granule/ISR signaling; ↑ ER protein load in ERO1-deficient cells; ↓ ATF4-mediated metastasis	Downstream ISR modulator; may reverse stress-induced drug tolerance and enhance chemotherapy or targeted therapy in selected models	May interfere with adaptive stress responses in normal tissues or reduce ER stress-induced tumor cell death in some contexts	Preclinical
ISRIB	PDAC	suppresses tumor growth; restore Gem-induced apoptosis (16, 126)	↓BZW1/PERK/eIF2α phosphorylation; ↓ISR activation			
ISRIB	HCC	Enhances sorafenib sensitivity; Synergizes with nelfinavir (127, 128)	↓ISR activation; selective ER stress response			
ISRIB	AML	Overcome chemoresistance; attenuates the growth suppression and apoptosis induced by the usnic acid + ABT-199 combination (129–131)	↓ISR and ATF4;↓ATF4-driven *ABCB1* upregulation; mRNA splicing correction			
ISRIB	CML	Synergizes with imatinib to eradicate TKI-resistant cells (132)	↓ RAS/RAF/MAPK and STAT5 signaling			
ISRIB	MM	reduces apoptosis induced by EGCG and 2P-Im (133, 134)	Attenuates UPR activation			
HC4	Metastatic Cancers	Eliminates dormant cancer cells; suppresses micrometastasis (135)	Disrupts PERK-dependent stress adaptation	Clinically advanced oral PERK inhibitor; may target dormant tumor cells, metastatic relapse, and antiangiogenic therapy resistance	Broader efficacy, long-term safety, and predictive biomarkers remain to be validated	Phase I; NCT04834778
HC-5404	RCC	Enhances anti-angiogenic therapy (VEGFR-TKI) (136)	↓PERK activation			
AMG-44	PC	Enhances Ref-1 inhibitors cytotoxic effects (137)	↓ISR	PERK/ISR-targeting agent that may strengthen oxidative stress- or Ref-1-targeted therapy	Limited pharmacokinetic, toxicity, and clinical translation data	Preclinical
IP6	CRC	Overcomes oxaliplatin resistance (138)	↓PERK pathway and eEF2 diphthamide modification	May reverse platinum resistance through PERK-associated translational control	Mechanism and patient selection require further validation	Preclinical

AML, acute myeloid leukemia; BC, breast cancer; CML, chronic myeloid leukemia; CRC, colorectal cancer; GBM, glioblastoma; HCC, hepatocellular carcinoma; MM, multiple myeloma; OC, ovarian cancer; OS, osteosarcoma; PC, pancreatic cancer; PDAC, pancreatic ductal adenocarcinoma; RCC, renal cell carcinoma; TNBC, triple-negative breast cancer. ↑ indicates increased or upregulated; ↓ indicates decreased or downregulated.

#### 3.2.1 GSK2606414

GSK2606414, a potent PERK inhibitor, exhibits broad antitumor activity by modulating ER stress and drug resistance (139), yet paradoxically promotes tumor survival in select contexts. *In vitro* studies indicate that GSK2606414 can overcome therapy resistance in several cancer models, including pancreatic ductal adenocarcinoma (110), breast cancer (107), colorectal cancer (112, 113), and leukemia (119), by disrupting PERK-associated adaptive stress pathways. Its antimigratory effects in medulloblastoma via VEGF-A/VEGFR2 suppression further highlight multimodal mechanisms (118). GSK2606414 also enhances the efficacy of several antitumor treatments, including metformin in ovarian cancer (108), folate-deficiency-induced apoptosis in hepatocellular carcinoma (115), and temozolomide/simvastatin in glioblastoma models (116). *In vivo*, GSK2606414 combined with radiotherapy suppresses senescence-associated recurrence in glioblastoma models (117). In pancreatic ductal adenocarcinoma, GSK2606414 remodels the tumor stroma by suppressing cancer-associated fibroblast transformation and enhances gemcitabine response (111). Leukemia models demonstrate strong synergy with digoxin (120). Paradoxically, GSK2606414 antagonizes ER stress-mediated therapies in specific contexts, such as blocking EVO-induced apoptosis in ovarian cancer (109), reversing the antitumor effects of the curcumin derivative WZ35 in colorectal cancer (114) and attenuating CYT997-induced apoptosis in osteosarcoma (121). Collectively, these findings highlight the multifaceted role of GSK2606414 in targeting the PERK pathway across various tumor types. By modulating ER stress, autophagy, and drug resistance, GSK2606414 emerges as a promising therapeutic strategy, either alone or in combination with other treatments, for improving cancer outcomes.

#### 3.2.2 ISRIB

The integrated stress response (ISR) aids tumor cell survival under adverse microenvironmental conditions by modulating ER stress. The ISR inhibitor ISRIB, a small molecule that allosterically antagonizes the inhibitory effect of phosphorylated eIF2 on eIF2B, has shown promise in alleviating ER stress and enhancing the efficacy of antitumor therapies (122, 140), yet paradoxically promotes tumor survival in select contexts. *In vitro* studies demonstrate that ISRIB enhances therapeutic sensitivity in several cancer contexts, including acute myeloid leukemia (129, 130) and hepatocellular carcinoma (127), by restoring drug responsiveness, limiting ATF4-dependent ABCB1-mediated chemoresistance, and improving sorafenib response. In addition, ISRIB inhibits doxorubicin-induced migration by disrupting mTORC1-eIF2α crosstalk (141). Paradoxically, ISRIB can also attenuate therapy-induced apoptosis in acute myeloid leukemia and myeloma models (131, 133, 134). *In vivo* applications reveal broader therapeutic potential. In triple-negative breast cancer, ISRIB-based strategies enhance doxorubicin sensitivity (123), synergize with ERO1 deficiency (122), suppress ETHE1-driven metastasis via ATF4 downregulation (124), and reduce primary/metastatic tumor growth when combined with nanodrug-photothermal therapy (125). In pancreatic ductal adenocarcinoma, BZW1 serves as a predictive biomarker for PERK-eIF2α inhibitors (16). Combinatorial targeting of this axis with GSK2606414 or ISRIB suppresses BZW1-driven tumor growth and prolongs survival (16), while ISRIB monotherapy reverses gemcitabine resistance (126). ISRIB combined with nelfinavir suppresses hepatocellular carcinoma progression (128), and ISRIB also synergizes with imatinib in tyrosine kinase inhibitor-resistant chronic myeloid leukemia models (132). Collectively, ISRIB has shown promising therapeutic potential across various cancers by enhancing drug sensitivity and inhibiting tumor progression. However, its efficacy is limited by complex resistance mechanisms, such as those in acute myeloid leukemia and myeloma, highlighting the need for further optimization.

#### 3.2.3 Other inhibitors

Recently, significant progress has been made in the development of novel PERK inhibitors for cancer therapy. HC4 disrupts the tumor microenvironment to eliminate dormant cancer cells and suppresses metastasis when combined with CDK4/6 inhibitors (135). Similarly, HC-5404 synergizes with VEGFR-TKIs to block PERK-mediated adaptive stress responses and enhance antiangiogenesis in renal carcinoma, leading to significant tumor growth inhibition (136). AMG-44 synergizes with Ref-1 inhibitors to enhance cytotoxicity against pancreatic cancer cells and CAFs (137). Moreover, inositol hexaphosphate (IP6) reverses oxaliplatin resistance in colorectal cancer by inhibiting PERK-mediated eEF2 diphthamide modification, thereby overcoming ER stress-induced senescence (138). Collectively, these novel PERK inhibitors show promising therapeutic efficacy in targeting dormant cancer cells, suppressing metastasis, and enhancing chemotherapy sensitivity.

Despite these promising preclinical findings, most PERK/ISR-targeting agents, including GSK2606414, ISRIB, AMG-44, and IP6, remain largely preclinical as cancer therapeutics. HC-5404/HC4 represents an important exception, as this oral PERK inhibitor has completed a registered Phase 1 trial in patients with advanced solid tumors, including renal cell carcinoma and gastric cancer (NCT04834778). Preliminary clinical findings indicate acceptable safety, dose-dependent exposure, pharmacodynamic pathway engagement, and early antitumor activity, supporting further evaluation of HC-5404 alone or in combination with antiangiogenic VEGFR tyrosine kinase inhibitors. Nevertheless, broader clinical translation of PERK/ISR-targeted therapies remains limited by potential proteostasis-related toxicity, pathway compensation, and the context-dependent effects of PERK inhibition.

### 3.3 ATF6-targeted therapeutics

Compared with the IRE1α and PERK branches, therapeutic strategies targeting ATF6 remain less developed. Nevertheless, accumulating evidence indicates that ATF6 signaling can be pharmacologically manipulated through several approaches, including direct inhibition of ATF6α, blockade of ATF6 processing, modulation of upstream regulators, and pharmacological activation of ATF6. Among currently available compounds, Ceapins are the first selective inhibitors of ATF6α and represent the most extensively characterized pharmacological tools targeting this pathway (142). Ceapins inhibit ATF6α activation in response to ER stress while exerting minimal effects on unstressed cells. Importantly, they selectively suppress ATF6α signaling without significantly affecting the IRE1α or PERK branches, ATF6β processing, or SREBP activation, highlighting their utility for both mechanistic studies and the development of branch-specific UPR-targeted therapies (142).

The antitumor potential of pharmacological ATF6 inhibition has been supported by several preclinical cancer models. Genetic depletion of ATF6 or pharmacological inhibition with Ceapin-A7 suppresses colorectal cancer cell proliferation, impairs cell-cycle progression, and inhibits tumor growth *in vivo*. Notably, ATF6 inhibition selectively reduces the growth of malignant intestinal organoids while largely sparing normal organoids, suggesting a potential therapeutic window for ATF6-targeted intervention (62). Mechanistically, ATF6 supports colorectal cancer progression by sustaining Wnt/MYC signaling and stemness-associated transcriptional programs that promote tumor growth and survival (62). Furthermore, STK26 has been identified as an upstream regulator of ATF6 signaling in colorectal cancer. STK26 is aberrantly upregulated in colorectal cancer and promotes tumor cell growth, proliferation, and migration by interacting with p50ATF6 and enhancing its protein stability. These STK26-driven phenotypes were attenuated by Ceapin-A7, while the STK26 inhibitor Hesperadin significantly suppressed colorectal cancer growth *in vivo*, supporting the STK26–ATF6 axis as a potential therapeutic target in colorectal cancer (143). Evidence from uterine cancer models further supports the direct antitumor activity of ATF6 inhibition, as Ceapin-A7 suppresses NHE7-driven ER stress signaling, proliferation, and invasion while promoting apoptosis (144). Beyond direct effects on tumor cells, Ceapin-A7 inhibits sFRP2-mediated endothelial tube formation and promotes endothelial apoptosis, indicating a potential role in suppressing tumor angiogenesis (145).

Despite these encouraging findings, current evidence also highlights important limitations and context-dependent effects of ATF6 inhibition. In colorectal cancer models, inhibition of ERO1L induces ER stress and sensitizes tumor cells to UPR-targeting agents. Under ERO1L-deficient ER stress conditions, Ceapin-A7 and GSK2606414 significantly reduced colorectal cancer cell viability, whereas 4μ8C did not produce a comparable effect (113). By contrast, Ceapin-A7 reversed IVA-induced RAD51 degradation and FBXO24 upregulation, thereby attenuating the synergistic antitumor activity of IVA combined with the PARP inhibitor olaparib (146). In an endometrial carcinoma model using Ishikawa cells exposed to insulin and palmitate, comparative inhibition of the three UPR branches demonstrated that pretreatment with the PERK inhibitor ISRIB markedly abolished insulin/PA-induced GRP78 upregulation and reduced CHOP expression, whereas the ATF6 inhibitor Ceapin-A7 showed limited effects on these ER stress markers (147). This result suggests that insulin plus PA treatment predominantly activates the PERK–CHOP arm of the UPR in endometrial carcinoma cells, making ATF6 inhibition less effective in this PERK-dominant stress context (147). Likewise, in lipid metabolic stress models, Ceapin-A7 only partially reduces CILP2-associated triglyceride accumulation and appears less potent than IRE1α inhibition, indicating that ATF6 may play a secondary role in certain metabolic stress responses (148). In lymphoma-related studies, Ceapin-A7 has primarily been used as a pathway-specific research tool rather than as a standalone therapeutic agent, emphasizing the importance of identifying tumors with genuine ATF6 dependency before clinical translation (149). In addition to context-dependent efficacy, indirect ATF6 inhibition is limited by target selectivity. Because ATF6 activation requires S1P-mediated proteolytic cleavage in the Golgi apparatus, the S1P inhibitor PF-429242 can reduce ATF6 and GRP78 expression and enhance susceptibility to ER stress-induced cell death (150). However, because S1P also regulates SREBP processing, PF-429242 lacks specificity for ATF6 and may influence lipid metabolism while triggering compensatory activation of the IRE1α and PERK pathways (150). Therefore, although indirect inhibition provides valuable mechanistic insights, it is generally considered less selective than direct inhibition by Ceapins.

Overall, ATF6-targeted therapies remain at the preclinical stage, with Ceapin-A7 and related Ceapins representing the most selective ATF6α inhibitors currently available. Existing evidence suggests that ATF6 inhibition may be most relevant in tumors with ATF6-dependent proteostasis, Wnt/MYC signaling, angiogenic remodeling, invasive phenotypes, or defined ER-stress-adaptive states. However, no registered clinical trial has yet been identified for Ceapin-A7 or other selective ATF6 inhibitors, and current evidence remains confined to mechanistic and preclinical cancer models. Therefore, clinical translation of ATF6 inhibitors will require the development of drug-like Ceapin derivatives, systematic *in vivo* pharmacokinetic and toxicity profiling, predictive biomarkers of ATF6 dependency such as cleaved ATF6α or ATF6 transcriptional signatures, and rational combination strategies to overcome compensatory activation of IRE1α or PERK.

### 3.4 Chemical chaperones

Chemical chaperones are small molecule compounds that facilitate the correct folding of proteins by assisting in the folding process or stabilizing the existing folded state, thereby reducing ER stress caused by protein misfolding. In tumor cells, ER stress has a dual role: it can either promote cell survival or trigger cell death. Similarly, chemical chaperones may either suppress tumor-promoting adaptive ER stress or protect tumor cells from therapies that rely on proteotoxic stress to induce cell death. Therefore, their therapeutic relevance should be interpreted according to tumor type, treatment context, and whether ER stress functions primarily as a survival mechanism or a death trigger (**Table 3**).

**Table 3:** Chemical chaperones in cancer therapy.

Inhibitor	Cancer type	Effects	Mechanisms	Advantages	Limitations	Clinical status
4-PBA	PDAC	reverses RUNX1-induced gemcitabine resistance (110)		Clinically familiar chemical chaperone; broadly reduces ER stress and can clarify whether tumor phenotypes depend on adaptive UPR activation	Non-branch-selective ER stress modulator; may also protect tumor cells from ER stress-induced cytotoxicity; anticancer efficacy is highly context dependent	NCT00001565, NCT00006450, and NCT00387530
4-PBA	HCC	Reduces ML-323-induced autophagy and apoptosis; promotes cancer stem cells (151, 152)	↑ PPAR-α/β-catenin axis			
4-PBA	CRC	Reduces p20BAP31-induced apoptosis (25)	↓ PERK/ROS pathway			
4-PBA	LUAD	Attenuates IFN-γ-induced apoptosis (153)	Restores LAMP1/2 expression and autophagy			
4-PBA	NSCLC	Reduces gambogic acid induced death (154)	↓ROS-induced ER stress			
4-PBA	gliomas	Attenuates xanthatin -induced apoptosis and autophagy (155)	↓CHOP-mediated ER stress pathway			
4-PBA	PCa	Reduces gambogenic acid induced death (156)	↓ JNK pathway			
4-PBA	teratoma	inhibits metastasis (157)	↑differentiation defects			
TUDCA	HCC	Inhibits Mst1/2 mutations-driven tumorigenesis (158)	↓ UPR pathway, ↑ YAP cytoplasmic degradation; ↓ ER stress	Broad ER stress modulator with translational relevance; may suppress tumorigenesis or metabolic stress adaptation in selected contexts	Non-specific UPR attenuation; may be cytoprotective and reduce therapy-induced apoptosis in some tumors; careful patient and combination selection is required	NCT07064668
TUDCA	DLBCL	Reduces ibrutinib-induced apoptosis (159)				

CRC, colorectal cancer; DLBCL, diffuse large B-cell lymphoma; HCC, hepatocellular carcinoma; LUAD, lung adenocarcinoma; NSCLC, non-small cell lung cancer; PCa, prostate cancer; PDAC, pancreatic ductal adenocarcinoma. ↑ indicates increased or upregulated; ↓ indicates decreased or downregulated.

#### 3.4.1 4-PBA

4-Phenylbutyric acid (4-PBA), an ER stress inhibitor, demonstrates context-dependent duality in cancer biology, exerting both tumor-suppressive and tumor-promoting effects across malignancies. *In vitro*, it reverses RUNX1-mediated gemcitabine resistance in pancreatic ductal adenocarcinoma (110) and attenuates IFN-γ-induced apoptosis in lung adenocarcinoma by restoring LAMP-1/LAMP-2 expression and autophagy (153). Paradoxically, 4-PBA protects tumor cells in multiple cancers by reducing ML-323-induced autophagy/apoptosis in hepatocellular carcinoma (151), alleviating p20BAP31-induced cell death in colorectal cancer (25), diminishing the proapoptotic effects of xanthatin in gliomas (155), and blocking gambogenic acid-induced JNK activation in prostate cancer (156). It also antagonizes the antitumor effects of gambogic acid in non-small cell lung cancer by mitigating ROS-dependent ER stress (154). *In vivo*, 4-PBA suppresses teratoma metastasis via ER stress reduction (157), and its salt form sodium phenylbutyrate inhibits oral squamous cell carcinoma progression by blocking TGF-β-mediated EMT (160). Conversely, in hepatocellular carcinoma models, it activates PPAR-α/β-catenin signaling to enrich cancer stem cells, accelerating tumor progression (152). Collectively, 4-PBA should not be considered a uniformly antitumor agent; its therapeutic value depends on whether ER stress is tumor-promoting or treatment-induced and cytotoxic.

#### 3.4.2 TUDCA

Tauroursodeoxycholic acid (TUDCA) is another broad ER stress modulator with context-dependent effects in cancer. *In vitro* studies reveal that the related bile acid ursodeoxycholic acid (UDCA), a precursor of TUDCA, synergizes with bortezomib in glioblastoma models (161). However, it also partially reverses ibrutinib-induced apoptosis in diffuse large B-cell lymphoma (159). *In vivo*, TUDCA suppresses Mst1/2 mutation-driven hepatocellular carcinoma by attenuating UPR and promoting Yap degradation (158), ameliorates colorectal cancer-associated cachexia through muscle/organ preservation without tumor acceleration (162), and enables tumor-targeted doxorubicin delivery via P-selectin-guided nanoparticles (t-pHUDs) to enhance efficacy while reducing toxicity (163). Collectively, TUDCA should be viewed as a nonspecific ER stress-modulating agent with context-dependent oncology relevance rather than as a broadly applicable anticancer therapy.

Although chemical chaperones are not branch-specific UPR inhibitors, the two agents discussed here—4-PBA and TUDCA—have been examined in registered oncology-related trials, providing a direct clinical reference point for broad ER stress modulation in cancer. 4-PBA has been tested in early-phase studies involving pediatric refractory malignancies and recurrent pediatric brain tumors (NCT00001565 and NCT00006450), while a trial of phenylbutyrate plus valganciclovir for relapsed or refractory EBV-positive cancer was registered but withdrawn (NCT00387530). TUDCA is also being prospectively evaluated in combination with PD-1/PD-L1 immunotherapy in advanced hepatocellular carcinoma (NCT07064668), suggesting that ER stress modulation may have translational relevance in cancer immunotherapy. However, because chemical chaperones broadly reduce ER stress rather than selectively inhibiting a single UPR branch, their clinical application will require careful patient stratification to determine whether ER stress attenuation suppresses tumor-promoting adaptation or inadvertently protects tumor cells from therapy-induced proteotoxic damage.

## 4 UPR signaling in tumor immunity and immunotherapy resistance

Beyond tumor-cell-intrinsic stress adaptation, UPR signaling also shapes antitumor immunity by modulating both malignant cells and immune components within the tumor microenvironment. Persistent ER stress can impair effector T-cell function, promote immune checkpoint expression, disrupt antigen presentation, and reprogram myeloid and dendritic-cell activity, thereby contributing to immune escape and resistance to immunotherapy. This section summarizes how UPR signaling influences T-cell dysfunction, immune checkpoint regulation, antigen presentation, and therapeutic responses to cancer immunotherapy.

### 4.1 T-cell dysfunction

T-cell activation and effector function require tight regulation of ER proteostasis, making UPR signaling an important determinant of T-cell fitness. Physiologically, balanced UPR activity is essential for T-cell survival, as deletion of the ERAD adaptor SEL1L induces unresolved ER stress, activates the PERK–ATF4–CHOP pathway, and impairs CD8^+^ T-cell homeostasis (164). However, persistent ER stress within the tumor microenvironment promotes T-cell dysfunction. Ma et al. demonstrated that cholesterol accumulation in tumor-infiltrating CD8^+^ T cells activates the IRE1α–XBP1 pathway, leading to increased expression of inhibitory receptors such as PD-1 and 2B4 and driving T-cell exhaustion (165). In parallel, nutrient deprivation and metabolic stress activate PERK–eIF2α signaling, suppress global protein translation, and limit cytokine production and effector functions in tumor-infiltrating T cells (166). Tumor-cell-intrinsic UPR activation may further reinforce this immunosuppressive state. PERK inhibition in tumor cells enhances immunogenic cell death, dendritic-cell activation, and antitumor T-cell responses, suggesting that aberrant tumor UPR activation can indirectly impair T-cell-mediated immunity (27). Recent studies further indicate that maintenance of proteostasis is associated with improved functionality of tumor-infiltrating lymphocytes, whereas disruption of protein quality-control networks accelerates T-cell exhaustion (167). Notably, pharmacological targeting of the GADD34–IRE1α signaling axis has been reported to alleviate CAR-T-cell exhaustion and improve antitumor efficacy. Mechanistically, GADD34 directly regulates IRE1α activity through a noncanonical pathway independent of its classical role in eIF2α dephosphorylation (168). Collectively, these findings indicate that physiological UPR signaling supports T-cell homeostasis, whereas chronic activation of the IRE1α–XBP1 and PERK–eIF2α pathways promotes T-cell dysfunction and exhaustion, thereby limiting antitumor immunity.

### 4.2 Immune checkpoint regulation

UPR signaling regulates immune checkpoint pathways through both tumor-cell-intrinsic stress adaptation and immune-cell dysfunction, thereby facilitating immune evasion and influencing the efficacy of immune checkpoint blockade (ICB) therapies. In hepatocellular carcinoma, persistent activation of ATF6α induces ER stress, promotes glycolytic metabolism, suppresses FBP1 expression, and establishes a nutrient-deprived immunosuppressive microenvironment that contributes to T-cell exhaustion; notably, ICB treatment can restore antitumor immune surveillance and reduce tumor burden in ATF6α-driven models (169). In tumor-infiltrating CD8^+^ T cells, cholesterol accumulation activates the IRE1α–XBP1 signaling pathway and upregulates inhibitory receptors such as PD-1, 2B4, TIM-3, and LAG-3, suggesting that UPR activation directly promotes an exhausted T-cell phenotype characterized by elevated checkpoint expression (165). In triple-negative breast cancer, the ER chaperone GRP78 interacts with PD-L1 and stabilizes its protein expression, providing a mechanism by which ER stress enhances tumor immune escape (170). In high-grade serous ovarian cancer, tumor-associated neutrophils exhibit enhanced ER stress and activation of the IRE1α pathway, which suppresses early T-cell-mediated antitumor immunity; importantly, neutrophil-specific deletion of IRE1α delays tumor progression and markedly sensitizes tumors to PD-1 blockade, resulting in tumor regression and prolonged survival in treated mice (39). In pancreatic ductal adenocarcinoma, cTRIP12 acts as a scaffold linking OGT and PERK signaling, thereby promoting O-GlcNAcylation-dependent upregulation of ferritin heavy chain and PD-L1 expression; moreover, pharmacological inhibition of PERK combined with ferroptosis induction and anti-CTLA-4 therapy significantly suppresses tumor growth in cTRIP12-high pancreatic ductal adenocarcinoma models (21). Collectively, these studies demonstrate that UPR signaling regulates immune checkpoint networks through multiple mechanisms, including ATF6α-mediated metabolic immunosuppression, XBP1-dependent induction of inhibitory receptors, GRP78-mediated stabilization of PD-L1, IRE1α-driven myeloid immunosuppression, and PERK-associated upregulation of PD-L1 expression.

### 4.3 Antigen presentation and immunotherapy resistance

UPR signaling also contributes to immunotherapy resistance by impairing antigen presentation and reducing the ability of CD8^+^ T cells to recognize tumor-derived antigens. In tumor-associated dendritic cells (tDCs), constitutive activation of XBP1 drives ovarian cancer progression by disrupting DC homeostasis and weakening T-cell-dependent antitumor immunity (171). Mechanistically, lipid peroxidation byproducts activate XBP1 in tDCs, inducing a triglyceride biosynthetic program that causes abnormal lipid accumulation and suppresses the capacity of DCs to support antitumor T-cell responses. Importantly, DC-specific deletion or nanoparticle-mediated silencing of XBP1 restores the immunostimulatory function of tDCs and prolongs survival by inducing protective type 1 antitumor immunity (171). Recent evidence further demonstrates that activation of the IRE1α–RIDD pathway in dendritic cells impairs antigen cross-presentation by degrading transcripts encoding MHC-I antigen-presentation machinery, thereby limiting CD8^+^ T-cell priming and antitumor immunity. Consistently, inhibition of IRE1α enhances MHC-I expression, promotes T-cell activation, and improves responses to immune checkpoint blockade in tumor-bearing mice (172). However, the immunological effects of IRE1α–XBP1 signaling in dendritic cells are not uniform across tumor models. Deletion of IRE1 RNase activity or XBP1s in DCs and cDC1s does not consistently enhance antitumor T-cell responses in melanoma and colon cancer models, underscoring the cell-type- and context-dependent role of this pathway in antigen presentation and T-cell priming (40). Tumor-cell-intrinsic IRE1α signaling also contributes to immune resistance, as genetic or pharmacological inhibition of IRE1α in prostate cancer activates interferon-related immune pathways, remodels the tumor microenvironment, reduces tumor-associated macrophage abundance, and enhances anti-PD-1 therapy (86). Beyond the IRE1α–XBP1 axis, ER-associated molecules involved in protein quality control and stress adaptation may also influence antigen-presentation programs and therapeutic resistance. For example, ORMDL2 expression in glioblastoma is associated with MHC-I antigen-presentation pathways, UPR signaling, immune suppression, poor prognosis, and resistance to DNA-damaging agents (173). Similarly, PDIA1 is linked to ER stress regulation and tumor immunorecognition, and its expression influences surface levels of the non-classical antigen-presenting molecule HLA-G under oxidative stress in breast cancer cells (174). Conversely, ERAD-related antigen-targeting strategies may enhance antitumor immunity, as fusion of the HPV E7 antigen to a COX-2-derived ERAD cassette elicits antigen-specific antitumor effects in prophylactic and therapeutic cancer models (175). Collectively, these findings indicate that UPR signaling regulates antigen presentation through multiple mechanisms, including XBP1-driven tDC lipid dysfunction, IRE1α/RIDD-mediated degradation of MHC-I transcripts, tumor-intrinsic IRE1α-dependent immune suppression, and ER-associated antigen processing, thereby contributing to impaired tumor immune recognition and resistance to cancer immunotherapy.

## 5 Future perspectives and challenges

Future research should shift from viewing UPR signaling as a broad stress-response network toward defining actionable UPR dependencies within specific tumor contexts (1, 6, 7). This shift is particularly important because PERK, IRE1α, and ATF6 function as context-dependent stress sensors rather than uniformly oncogenic drivers, and their biological outputs are shaped by tumor genotype, stress intensity, immune contexture, and therapeutic pressure (1, 2, 6, 7). Moderate activation of the UPR can promote tumor survival, metastasis, immune evasion, and therapeutic resistance, whereas excessive or unresolved ER stress may redirect the same signaling network toward apoptosis, ferroptosis, immunogenic cell death, or inflammatory tumor suppression (1, 7, 20, 26–28). Moreover, the three canonical UPR branches are highly interconnected, and inhibition of one pathway may trigger compensatory activation of another, thereby reducing the long-term efficacy of single-pathway interventions (63, 65, 66). Therefore, future development should focus on converting pathway-specific biological insights into clinically actionable strategies through targeted protein degradation, nanomedicine-based delivery, biomarker-guided patient selection, and rational combination therapy (1, 3, 6, 7, 176). These approaches may help overcome key barriers that currently limit UPR-targeted therapy, including pathway compensation, systemic toxicity, context-dependent biological effects, and the lack of validated predictive biomarkers (2, 6, 65, 66).

### 5.1 Targeted protein degradation

Targeted protein degradation technologies, including proteolysis-targeting chimeras and related degrader platforms, represent a promising next-generation approach for UPR modulation by eliminating disease-associated proteins rather than merely inhibiting their enzymatic activity (176). This strategy may be particularly relevant for IRE1α, as accumulating evidence suggests that IRE1α contributes to cancer cell-cycle progression and tumor growth through nonenzymatic functions that cannot be fully blocked by conventional inhibitors (37). Therefore, an IRE1α-directed degrader could theoretically suppress both RNase-dependent activities, such as XBP1 splicing and RIDD, as well as nonenzymatic scaffolding functions that support tumor progression (3, 37, 176). Similar degradation-based approaches may also be explored for PERK or ATF6 in tumors exhibiting branch-specific dependence. However, systemic depletion of these ER stress sensors may raise safety concerns because UPR signaling is essential for maintaining proteostasis in normal secretory tissues and immune cells (2, 6, 164). Although targeted degradation of UPR components remains largely at the conceptual and preclinical stage, it offers a rational strategy to overcome incomplete target inhibition and compensatory pathway activation.

### 5.2 Nanomedicine-based delivery systems

Nanomedicine-based delivery systems may help overcome one of the major limitations of UPR-targeted therapies, namely systemic toxicity resulting from inhibition of adaptive stress pathways that are also required in normal tissues (2, 6). A compelling proof-of-concept was demonstrated in ovarian cancer models, where nanoparticle-mediated silencing of XBP1 in tumor-associated dendritic cells restored their immunostimulatory function and prolonged survival by promoting protective type I antitumor immunity (171). This finding highlights the potential of nanomedicine not only as a drug delivery platform but also as a means of selectively reprogramming ER stress signaling within specific cellular compartments of the tumor microenvironment (171). Nanotechnology may also improve the delivery of small-molecule UPR modulators. For example, hollow copper sulfide nanoparticles loaded with ISRIB enhanced photothermal therapy in breast cancer and brain metastasis models by suppressing stress granule formation and reprogramming tumor-associated macrophages (125). Moreover, tumor-targeted and stimulus-responsive polymeric prodrug nanoparticles incorporating a TUDCA-related platform improved doxorubicin delivery while reducing systemic toxicity, supporting the feasibility of combining ER stress modulation with targeted nanotherapeutics (163). Future nanomedicine strategies should prioritize cell-specific delivery to tumor cells, dendritic cells, macrophages, or T cells, given that the same UPR pathway may exert distinct or even opposing effects depending on the cellular context.

### 5.3 Biomarker-guided therapies and patient stratification

Biomarker-guided patient stratification will be critical for successful clinical translation because dependence on UPR signaling varies according to tumor type, genetic background, stress intensity, therapeutic pressure, and immune context (1, 6, 7). For IRE1α-targeted therapies, potential pharmacodynamic biomarkers include XBP1 splicing, RIDD activity, and downstream immune remodeling, as inhibition of IRE1α RNase activity directly influences XBP1s production, RIDD-mediated RNA degradation, and tumor–immune interactions (70). For PERK/ISR-targeted approaches, p-eIF2α, ATF4, CHOP, and downstream translational signatures may serve as useful pharmacodynamic indicators, while BZW1 has been proposed as a predictive biomarker for sensitivity to PERK–eIF2α pathway inhibition in pancreatic ductal adenocarcinoma (16). In addition to these canonical pathway markers, upstream regulators of PERK signaling may provide further opportunities for biomarker-guided patient selection. TRIM29 is particularly relevant in this context because it has been implicated in cancer progression (177) and can enhance PERK-mediated ER stress signaling by promoting PERK SUMOylation (14). Notably, the PERK inhibitor GSK2656157 disrupts the TRIM29–PERK axis, suggesting that pharmacological inhibition of this regulatory module may represent a potential therapeutic strategy for tumors characterized by TRIM29-dependent PERK activation (14). In the context of ATF6-targeted therapies, cleaved ATF6α, ATF6-associated transcriptional signatures, Wnt/MYC signaling activity, and stemness-related gene expression may help identify tumors that are particularly dependent on ATF6 signaling (62). Immune-related biomarkers should also be incorporated into future clinical trials because UPR signaling influences PD-L1 stabilization, antigen presentation, dendritic-cell dysfunction, myeloid-cell polarization, and T-cell exhaustion (164–167, 169–175). In this regard, TRIM29 may also have immunological relevance, as it negatively regulates RNA- and DNA-sensing-mediated type I interferon production, pathways that play critical roles in antiviral innate immunity and tumor immune surveillance (178–180). These findings raise the possibility that TRIM29-targeted strategies may affect both PERK-dependent stress adaptation and nucleic acid-sensing immune pathways, although their relevance to antitumor immunity requires further investigation. Accordingly, future studies should integrate paired tumor biopsies, UPR transcriptional profiling, single-cell analyses, immune microenvironment characterization, pharmacodynamic monitoring, and assessment of upstream regulatory markers such as TRIM29 expression, PERK SUMOylation status, and type I interferon-related signatures to identify patients most likely to benefit from UPR-targeted interventions.

### 5.4 Rational combination treatment strategies

Combination therapies are likely to be more effective than single-agent UPR inhibition because tumor cells can adapt to ER stress through pathway redundancy, metabolic reprogramming, and activation of parallel stress-response mechanisms (63, 65, 66). One promising approach is to combine UPR inhibitors with therapies that impose proteotoxic, metabolic, mitotic, or immune stress on tumor cells. For example, inhibition of IRE1α RNase activity enhances the antitumor efficacy of paclitaxel in triple-negative breast cancer by altering tumor-cell secretory programs (38). Furthermore, MKC8866 prevents taxane-induced degradation of double-stranded RNA through inhibition of the IRE1α–RIDD axis, thereby promoting ZBP1 activation, NLRP3–GSDMD-mediated pyroptosis, and immunogenic tumor-cell death (36). UPR-targeted agents may also be combined with molecularly targeted therapies; notably, MKC8866 enhances the efficacy of sotorasib and trametinib in KRAS-mutant lung and pancreatic cancer models (92). PERK inhibition may be particularly valuable in combination with antiangiogenic therapies, as HC-5404 sensitizes renal cell carcinoma models to VEGFR tyrosine kinase inhibitors by blocking PERK-mediated adaptive stress responses (136). UPR modulation may further improve immunotherapy outcomes. For instance, IRE1α inhibition reprograms the prostate cancer microenvironment, reduces tumor-associated macrophage infiltration, activates interferon-related immune pathways, and enhances responsiveness to anti-PD-1 therapy (85, 86). Similarly, B-I09 suppresses CARM1-expressing ovarian cancer and synergizes with anti-PD-1 treatment through inhibition of the IRE1α/XBP1s pathway (93). Combining UPR-targeted therapies with ferroptosis-inducing strategies may also be beneficial, but such combinations should be designed in a branch-specific manner. PERK signaling regulates SLC7A11-dependent antioxidant defenses and ferroptosis sensitivity in colorectal cancer (20), whereas IRE1α RNase activity promotes ferroptosis by cleaving and downregulating mRNAs encoding key glutathione biosynthesis regulators, including GCLC and SLC7A11 (181). Collectively, these findings support a future therapeutic paradigm in which UPR-targeted agents are combined with chemotherapy, targeted therapy, immunotherapy, antiangiogenic therapy, or ferroptosis induction according to the dominant stress-adaptation mechanisms operating within individual tumors.

### 5.5 Translational outlook

To date, only a limited number of UPR-targeted or UPR-modulating agents have entered clinical evaluation, and the clinical progress of representative inhibitors has been discussed in the corresponding sections of this review. Among branch-specific inhibitors, MKC8866 is currently the most advanced IRE1α RNase inhibitor in clinical development, whereas HC-5404/HC4 represents a notable PERK inhibitor that has entered clinical evaluation in patients with advanced solid tumors (3, 70, 71, 136). Chemical chaperones such as 4-PBA and TUDCA have been more widely investigated as ER stress-modulating agents; however, their nonspecific attenuation of ER stress introduces uncertainty because they may either suppress tumor-promoting adaptive responses or protect tumor cells from therapy-induced proteotoxic stress depending on the biological context (1, 7, 159–161). A major safety concern is that UPR signaling is indispensable for physiological proteostasis in secretory tissues and immune cells, meaning that systemic inhibition may result in tissue toxicity or compromise antitumor immunity (2, 6, 164). Another unresolved challenge is the dual role of PERK, IRE1α, and ATF6 in cancer, as each pathway can either promote or suppress tumor progression depending on stress intensity, genetic background, microenvironmental conditions, and treatment context (45, 46, 63, 65, 66). To address these challenges, future research should prioritize tumor-selective delivery systems, pharmacodynamic monitoring, biomarker-driven patient enrollment, and mechanism-based combination strategies rather than broad, nonspecific UPR inhibition (1, 3, 7, 70, 71). Overall, the integration of targeted protein degradation, nanomedicine-based delivery, biomarker-guided patient selection, and rational combination therapies may facilitate the transition of UPR-targeted interventions from promising preclinical concepts to precise and clinically actionable anticancer strategies.
